# Evening Primrose Oil Enhances Tamoxifen’s Anticancer Activity against Breast Cancer Cells by Inducing Apoptosis, Inhibiting Angiogenesis, and Arresting the Cell Cycle

**DOI:** 10.3390/molecules27082391

**Published:** 2022-04-07

**Authors:** Mohammad M. Abd-Alhaseeb, Sarah M. Massoud, Fatma Elsayed, Gamal A. Omran, Ahmad Salahuddin

**Affiliations:** 1 Department of Pharmacology & Toxicology, Faculty of Pharmacy, Damanhour University, Damanhour 22511, Egypt; m.abdelhasseb@pharm.dmu.edu.eg; 2 Department of Biochemistry, Faculty of Pharmacy, Damanhour University, Damanhour 22511, Egypt; s.masoud05563@pharm.dmu.edu.eg (S.M.M.); gamal.omran@pharm.dmu.edu.eg (G.A.O.); 3 Cell Culture Unit, Medical Research Institute, Alexandria University, Alexandria 21648, Egypt; elsayed_fatma2012@yahoo.com

**Keywords:** evening primrose oil, tamoxifen, MCF-7, MDA-MB-231, breast cancer

## Abstract

Background: Despite advancements in cancer treatment, breast cancer (BC) is still one of the leading causes of death among women. The majority of anti-breast-cancer medications induce serious side effects and multidrug resistance. Although several natural compounds, such as evening primrose oil (EPO), have been shown to have anticancer properties when used alone, their combination with the anticancer medicine tamoxifen (TAM) has yet to be investigated. The present study aimed to investigate the anticancer efficacy of EPO, alone or in combination with TAM, in the BC cell lines MCF-7 and MDA-MB-231, as well as to elucidate the mechanism of action. Methods: The MTT assay was used to investigate the cytotoxic effect of EPO on the two cell lines, and we discovered an acceptable IC_50_ that was comparable to TAM. The ELISA, qRT-PCR, flow cytometry and colorimetric techniques were used. Results: The combination of EPO and TAM suppressed the VEGF level, VEGF gene expression and Cyclin D1 signaling pathways, arrested the cell cycle, and induced the apoptotic signaling pathways by increasing the Bax/Bcl-2 ratio and caspase 3 activity; this revealed significant anti-tumor activity. Conclusions: The most significant finding of this study was the confirmation of the anticancer activity of the natural product EPO, which potentiated the activity of the anticancer drug TAM against MCF-7 and MDA-MB-231 BC cell lines through the induction of apoptosis, inhibiting angiogenesis and halting cell proliferation.

## 1. Introduction

Breast cancer (BC) is the most prevalent type of cancer in women. In 2020, there were around 2.3 million women diagnosed and 685,000 deaths globally [1]. Cancer cell lines have been extensively studied to investigate the molecular pathways behind cancer. Numerous BC cell lines are suitable for in vitro investigations because they preserve certain ideal properties of the mammary epithelium [2].

MCF-7 and MDA-MB-231 are examples of BC cell lines. MCF-7 is a non-invasive cell line that expresses estrogen receptors (ER), and progesterone receptors (PR) but does not express human epidermal growth factor receptor 2 (HER2); it is a model of early-stage breast cancer [3].

The MDA-MB-231 cell line is a model of triple-negative BC as it does not express ER, PR, nor HER2; rather, it expresses mutated p53. It is commonly used to model late-stage BC associated with metastasis [4].

α-ER plays a critical role in breast cancer carcinogenesis by upregulating cyclin D1, Myc, B-cell lymphoma 2 (Bcl-2) and vascular endothelial growth factor (VEGF), all of which are involved in the cell cycle, in cell survival and in angiogenesis stimulation [5].

Tamoxifen (TAM), an antiestrogenic drug, and is widely used to treat BC. TAM is suggested to have a cytotoxic and cytostatic effect on both ER-positive and ER-negative BC cell lines in vitro [6]. TAM is thought to have ER-dependent effects at low doses and ER-independent effects at higher doses. Thus, TAM may exhibit both ER-mediated and non-ER-mediated activity [7]. TAM is known to exert anticarcinogenic effects in breast and colon cancers via an ER-dependent mechanism, as well as in hepatocellular carcinoma via an ER-independent mechanism. Additionally, TAM is effective at reversing multidrug resistance in gastrointestinal carcinoma, colorectal carcinoma and cholangiocarcinoma by directly binding to and inhibiting the P-glycoprotein [8].

Unfortunately, TAM’s prolonged chemotherapeutic course is related to adverse events such as thromboembolism, endometrial hyperplasia and polyps, and secondary endometrial malignancy. These adverse consequences are mostly caused by TAM’s inability to differentiate between rapidly developing cancer cells and normal proliferating cells [9].

Natural products are increasingly being used to ameliorate human cancer with few adverse effects [10]. Evening primrose oil (EPO) is obtained through cold pressing or solvent extraction of the seeds of evening primrose (*Oenothera biennis*), a member of the Onagraceae family [11]. EPO contains high concentrations of linoleic acid (70–74%) and γ-linolenic acid (8–10%), which may contribute to the normal functioning of human tissues, because they are precursors to anti-inflammatory eicosanoids. EPO is well known for its beneficial effects on premenopausal, menopausal, postmenopausal, and mastalgia disorders [12,13]. EPO supplementation results in an increase in the plasma levels of γ-linolenic acid and its metabolite dihomo-γ-linolenic acid [14]. γ-linolenic acid has been shown to inhibit various cancer cells without affecting normal cells. γ-linolenic acid may exert its anticancer effect by altering the hypoxic microenvironment, mitochondria-mediated death, apoptosis, and anti-inflammatory pathways [15].

This research aims to determine the anticancer activity of EPO alone or in combination with TAM in the BC cell lines MCF-7 and MDA-MB-231, and to ascertain the mechanism of action.

## 2. Materials and Methods

### 2.1. Chemicals

The EPO purchased was Puritan’s Pride Cold-Pressed Evening Primrose Oil 1000 mg Softgels, from Puritan’s Pride, Inc. (Oakdale, NY, USA). The content of each softgel containing 1000 mg of EPO, according to the manufacturer, was 730 mg linoleic acid and 90 mg γ-linolenic acid.

TAM (CAS # 10540–29–1) was purchased from Sigma Chemical Co. (St. Louis, MO, USA). 3-(4, 5-dimethyl thiazolyl-2)-2, 5-diphenyltetrazolium bromide (MTT) was procured from Serva (Heidelberg, Germany). Dulbecco’s modified Eagle’s medium (DMEM), trypsin, phosphate-buffered saline (PBS), and a penicillin/streptomycin mixture were procured from Lonza^®^ (Basel, Switzerland).

All other chemicals and materials were commercially available and of standard quality.

### 2.2. Experimental Cell Line

Human breast adenocarcinoma MCF-7 and MDA-MB-231 were provided by the American Type Culture Collection (ATTC, Manassas, VA, USA). Cells were grown in DMEM with 1% penicillin/streptomycin and 10% FBS, and incubated at 37 °C with humidified air and 5% CO_2_.

### 2.3. Ethical Approval

This study was approved by the ethical committee of the Faculty of Pharmacy, Damanhour University (Ref. No. 719PB11).

### 2.4. Cell Viability Assay

The effects of EPO and TAM on cell viability were evaluated using an MTT assay. MCF-7 and MDA-MB-231 were separately seeded in a 96-well plate [1 × 10^4^ cells/well]. Each well contained 100 μL DMEM medium supplemented with 10% FBS, incubated at 37 °C for 24 h, under 5% CO_2_ and 95% air, until reaching 70–80% confluence.

The old media were discarded, then 0.1 mL of DMEM containing different drug concentrations was added and incubated for another 72 h.

- TAM concentrations (1, 0.5, 0.25, 0.125, and 0.0652 μg/mL) or EPO concentrations (250, 125, 62.5, 31.25, and 15.63 μg/mL) were added to all MCF-7 wells, except the control wells.
- TAM concentrations (8, 4, 2, 1, and 0.5 μg/mL) or EPO concentrations (250, 125, 62.5, 31.25, and 15.63 μg/mL) were added to all MDA-MB-231 wells except the control wells.

The media were then aspirated, and cells were incubated with 200 μL MTT working solution (0.5 mg/mL in DMEM) for four hours in the dark. After removing the supernatant, the resulting formazan crystals were solubilized in 100 μL of DMSO by maintaining agitation for 15 min. Absorbance was recorded at 590 nm using a microplate reader. All experiments were conducted at least three times independently, each performed in triplicate. The viability of the cells was calculated as a percentage relative to that of the control wells. The median inhibitory concentration (IC_50_) values were calculated using CompuSyn software (CompuSyn, Inc., version 1, Paramus, NJ, USA) [16].

### 2.5. Treatment of MCF-7 and MDA-MB-231 Cells for Biomolecular Investigations

Cells were seeded at 500,000 cells per T-25 flask containing complete media, and allowed to adhere overnight at 37 °C (15 flasks for MCF-7 cells and 15 flasks for MDA-MB-231 cells). The next day, cells were segregated into groups (3 flasks in each group), and treated as follows:

- Control: cells were treated with 1% DMSO, in both cell lines.
- EPO: cells were treated with (IC_50_) concentration of EPO, in both cell lines.
- TAM: cells were treated with (IC_50_) concentration of TAM, in both cell lines.
- Combination-1: cells were treated with 50% of (IC_50_) concentration of EPO +50% of (IC_50_) concentration of TAM, in both cell lines.
- Combination-2: cells were treated with (IC_50_) concentration of EPO + (IC_50_) concentration of TAM, in both cell lines.

All treatments were applied to MCF-7 and MDA-MB-231 cells at 70–80% confluence, and the cells were incubated in a CO_2_ incubator for 72 h; then, the cells were harvested and portioned into aliquots. Finally, the aliquots were kept at −80 °C for further investigations.

### 2.6. Preparation of Cell Lysates

Cell lysates were prepared with a RIPA lysis buffer (Boster Biological Technology, Pleasanton, CA, USA, Catalog # AR0105). Briefly, 0.5 mL of chilled RIPA lysis buffer was added to the cell pellet, vortexed, and incubated for 30 min on ice. Then, samples were centrifuged at 14,000× *g* for 10 min, and the supernatant was transferred to another tube for further analysis.

### 2.7. Protein Quantitation Using BCA Assay

Protein concentration was determined using the SMART^TM^ BCA protein assay kit (#21071) purchased from iNtRON Biotechnology, Inc. (Gyeonggi, Korea). The absorbance was read at 562 nm.

### 2.8. Biomarker Analysis Using ELISA Technique

VEGF and cyclin D1 were evaluated in the cell lysates from different treatment groups using the ELISA technique, using a human VEGF ELISA kit and a human cyclin D1 ELISA kit, supplied by MyBioSource inc. (San Diego, CA, USA). The manufacturer’s protocol was followed for all measurements. Each parameter was assayed in triplicate and was expressed relative to the total protein content in the same sample.

### 2.9. Caspase-3 Activity Assay

A colorimetric kit obtained from Sigma-Aldrich (St. Louis, MO, USA) was utilized to assess caspase-3 activity following the manufacturer’s procedure.

### 2.10. qRT-PCR Determination of mRNA Genes Expression of VEGF, Bax, and Bcl-2

Total RNA was isolated from MCF-7 and MDA-MB-231 using gene easy-RED^TM^ total RNA extraction Kit (iNtRON Biotechnology, Inc., Gyeonggi, Korea). RNA concentration and purity were determined using nanodrop (Q5000, Quawell, CA, USA) and 1% gel electrophoresis. RNA (5 μg) was reverse-transcripted using the Maxime RT Premix kit (iNtRON Biotechnology, Inc., Gyeonggi, Korea). The cDNA produced was used as a template to determine the relative expression of VEGF, Bax, and Bcl-2 genes using Maxime RT PreMix kit (iNtRON Biotechnology, Inc., Gyeonggi, Korea) and specific primers (Table 1). β-actin was used as an internal control. The thermal cycling conditions, melting curve temperatures and relative expression calculations were performed using 2^−ΔΔCt^.

### 2.11. Annexin V—FITC/PI Double Staining for Flow-Cytometric Apoptosis Detection

Cell apoptosis was assessed using an annexin V/FITC Apoptosis Detection Kit (Catalog #: K101–25, BioVision, Inc., Milpitas, CA, USA), according to the manufacturer’s instructions. Following trypsinization, MCF-7 and MDA-MB-231 cells from all studied groups were centrifuged to collect 5 × 10^5^ cells. Cell pellets were washed with cold 1X PBS and resuspended in 500 μL annexin-binding buffer. Then, 5 μL propidium iodide and 5 μL annexin V/FITC were added and incubated at room temperature for 5 min in the dark. Apoptotic cells were detected using Attune flow cytometer (Applied Biosystems, Foster City, CA, USA).

### 2.12. Cell-Cycle Analysis by Flow Cytometry

Following trypsinization, MCF-7 and MDA-MB-231 cells from all studied groups were centrifuged. Cell pellets were washed twice, resuspended in warm PBS, fixed using ice-cold absolute ethanol, and incubated for at least 24 h at −20 °C. Samples were centrifuged and the supernatant was removed. Pellets were homogenized in 5 mL cold PBS and centrifuged; 1 mL PBS-Triton X100 and 100 μL RNase-A (200 μg/mL) were added and incubated at 37 °C for 30 min; then, 100 μL propidium iodide (1 mg/mL) was added and incubated in darkness for 30–60 min at room temperature. Cell-cycle analysis was carried out using an Attune flow cytometer (Applied Biosystems, Foster City, CA, USA).

### 2.13. Characterization of EPO Using Gas Chromatography–Mass Spectrometry (GC-MS) Analysis

The chemical composition of EPO in the supplied softgel was analyzed using a Trace GC-TSQ mass spectrometer (Thermo Scientific, Austin, TX, USA) with a direct capillary column TG–5MS (30 m × 0.25 mm × 0.25 µm film thickness).

An amount of 0.35 g EPO was added to 6 mL 0.5 mol/L NaOH-methanol in a 250 mL flask, and it was shaken well. Then, 7 mL 12.5% BF_3_-methanol was added and it was shaken again. The mixture was refluxed at 70 °C for 2–3 min, then 5 mL hexane was added through the reflux system and heating continued for an additional 2 min. During reflux, the mixture was shaken occasionally until the methylation process was completed. After cooling, 30 mL saturated NaCl solution was added and extracted vigorously. The aqueous phase was transferred into a 250 mL separatory funnel and extracted with two 50 mL petroleum ethers. The combined organic layer was washed with Aqua Bidest until free of base, and the solvent was evaporated using a rotary evaporator. The concentrate was dissolved with 1 mL hexane prior to injection [17].

The column oven temperature was initially held at 100 °C for 1 min, then increased by 10 °C/min to 160 °C, then increased again to 220 °C with 2.5 °C/min, then increased once more to 240 °C with 10 °C/min. The injector temperature was kept at 270 °C. Helium was used as a carrier gas at a constant flow rate of 1 ml/min. The solvent delay was 4 min and diluted samples of 1 µL were injected automatically using an Autosampler AS3000 coupled with GC in split mode. EI mass spectra were collected at 70 eV ionization voltages over an *m*/*z* range of 50–650 in full-scan mode. The ion source and transfer line were set at 250 °C and 280 °C, respectively. The components were identified by comparing their mass spectra with those in the WILEY 09 and NIST 14 mass spectral database.

### 2.14. Statistical Analysis

Values were analyzed using Graph Pad Prism 8 (Graph Pad Software, Inc., San Diego, CA, USA) using one-way ANOVA, followed post hoc by Tukey’s multiple comparisons tests. The results were expressed as the mean ± standard deviation of the mean (SD). Significant differences among means were estimated at *p* < 0.05.

## 3. Results

### 3.1. GC-MS Analysis of EPO

**Figure 1:**
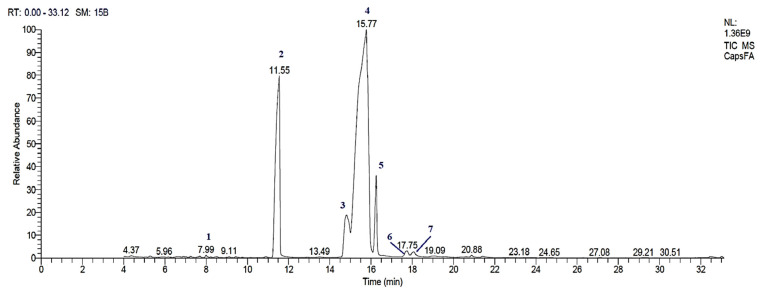
GC-MS Chromatographic profile of EPO: (1) methyl myristate; (2) methyl palmitate; (3) methyl γ-linolenate; (4) methyl linoleate; (5) methyl stearate; (6) 6,9-Octadecadienoic acid (E, E), methyl ester; (7) linoleic acid. EPO: evening primrose oil.

The GC-MS chromatogram of EPO in the supplied softgel showed seven peaks (Figure 1). The mass spectral fingerprint of each compound was identified from the data library. From the chromatogram peaks, different fatty acid methyl esters (FAMEs) were found, and the details of each compound are listed in Table 2. Methyl linoleate (43.05%), methyl palmitate (43.06%), methyl stearate (7.91%), and methyl γ-linolenate (7.32%) were identified as the major constituents of EPO.

### 3.2. Effect of EPO and TAM on MCF-7 and MDA-MB-231 Cell Viability

**Figure 2:**
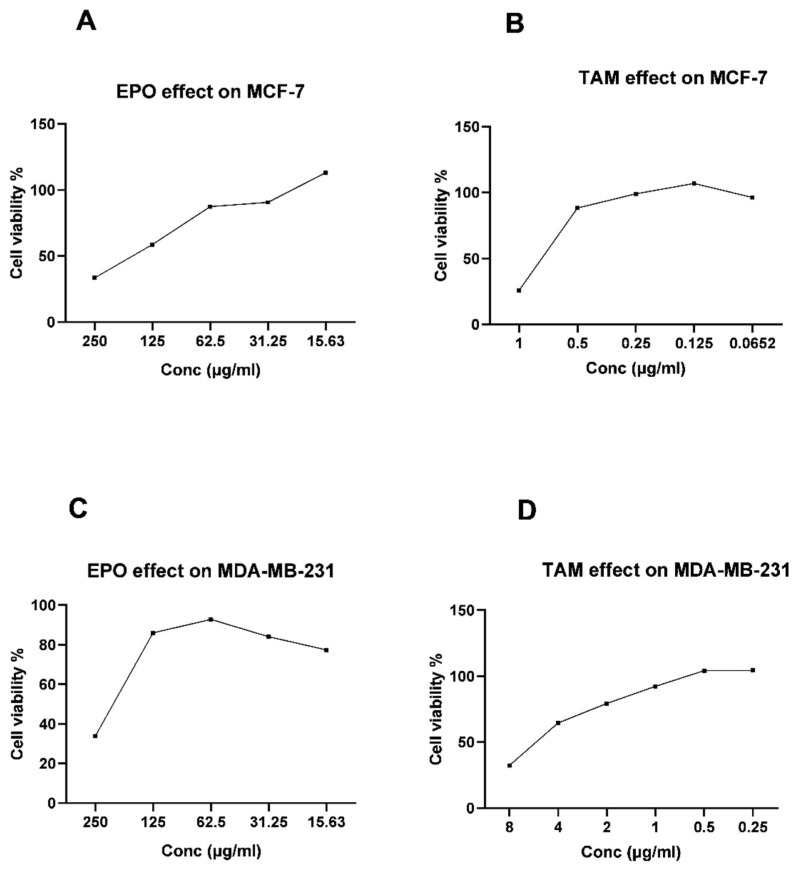
The viability of MCF-7 cells treated with EPO (**A**) and TAM (**B**); the viability of MDA-MB-231 cells treated with EPO (**C**) and TAM (**D**). EPO: evening primrose oil; TAM: tamoxifen.

The MTT assay results show significant dose-dependent antiproliferative activity for the EPO and TAM when used alone. TAM and EPO showed IC_50_ = 0.78 μg/mL and 183.96 μg/mL, respectively, on the MCF-7 cells, and showed IC_50_ = 5.92 μg/mL and 216.9 μg/mL, respectively, on MDA-MB-231 cells (Figure 2).

### 3.3. Effect of TAM and EPO, Alone or in Combination, on Caspase-3 Activity, VEGF and Cyclin D-1 Levels

**Figure 3:**
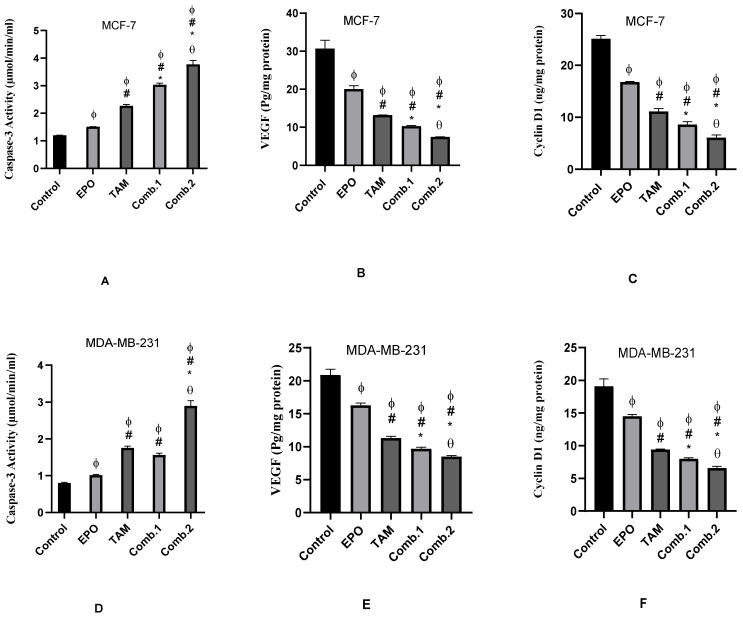
Effect of EPO and TAM, alone or in combination, on the activity of caspase-3 (µmol/mL/min—**A**,**D**), and on the levels of VEGF (pg/mg protein—**B**,**E**) and Cyclin D1 (ng/mg protein—**C**,**F**) in MCF-7 and MDA-MB-231 cells. Results are shown to be significant at *p* < 0.05. EPO: evening primrose oil; TAM: tamoxifen; ϕ: is significant from the control group; #: is significant from the EPO group; *: is significant from the TAM group; θ: is significant from the combination-1 group. (**A**–**C**) represent results in MCF-7 cells; (**D**–**F**) represent results in MDA-MB-231 cells.

EPO and TAM showed a significant increase in caspase-3 activity and a significant (*p* < 0.05) decrease in the levels of VEGF and cyclin-1 in the treated groups of both cell lines when compared to the control group. Additionally, there were significant (*p* < 0.05) differences between the combination groups and single-treated groups. Furthermore, the Combination-2 group showed significant differences compared to the combination-1 group (Figure 3).

### 3.4. Effect of TAM and EPO, Alone and in Combination, on VEGF, Bcl-2, and Bax Gene Expression

**Figure 4:**
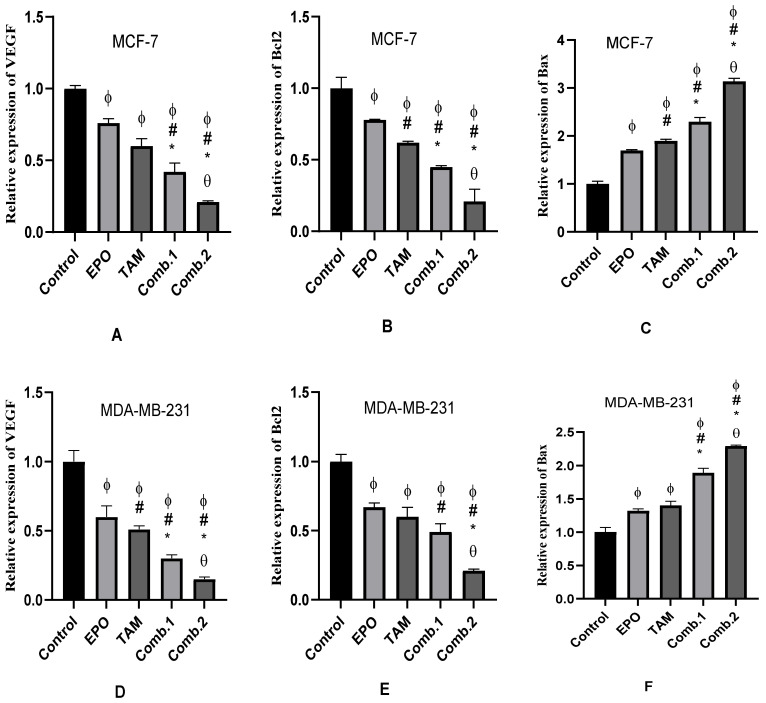
The expression of VEGF, Bcl2, and Bax genes induced by EPO and TAM, alone or in combination, in MCF-7 and MDA-MB-231 cells. Results are shown to be significant at *p* < 0.05. EPO: evening primrose oil; TAM: tamoxifen; ϕ: is significant from the control group; #: is significant from the EPO group; *: is significant from the TAM group; θ: is significant from the combination-1 group. (**A**–**C**) represents results in MCF-7 cells; (**D**–**F**) represents results in MDA-MB-231 cells.

The qRT-PCR results showed significant (*p* < 0.05) downregulation in the expression of VEGF and Bcl2 genes, and upregulation in the expression of Bax, in all treated groups when compared with the control. In addition, the combination groups showed significant changes when compared with the single-treated groups. Furthermore, these differences were significant between the combination-2 and combination-1 groups (Figure 4).

**Figure 5:**
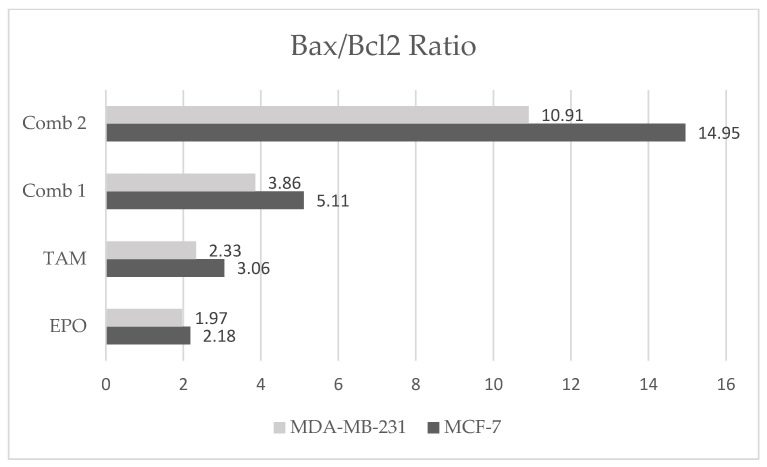
Effect of EPO and TAM, alone or in combination, on Bax/Bcl2 ratio in MCF-7 and MDA-MB-231 cells. EPO: evening primrose oil; TAM: tamoxifen.

In addition, the results showed an increase in Bax/Bcl2 ratio in the combination-2 group compared with the other treated groups in both cell lines, with a more notable increase in this ratio in the MCF-7 cell line than in the MDA-MB-231 cells (Figure 5).

### 3.5. Effect of TAM and EPO, Alone or in Combination, on Apoptosis

**Figure 6:**
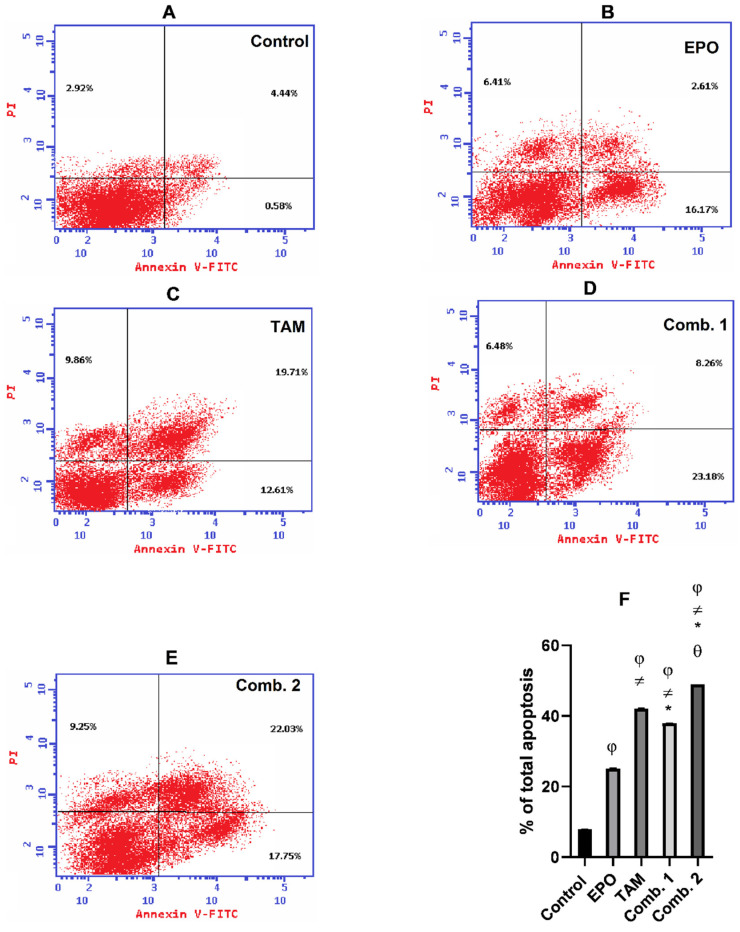
Effect of EPO and TAM, alone or in combination, on apoptosis in MCF-7 cells: (**A**–**E**) Dot-plots from flow-cytometric analysis of cell apoptosis using annexin V-FITC/PI dual-staining in MCF7 cells. Cells in the upper-left quadrant are necrotic, cells in the upper-right quadrant have late apoptosis, the lower-left quadrant has normal living cells, and the cells in the lower-right quadrant are early apoptotic cells; (**F**) the bar chart describes the total percentage of apoptosis in MCF7. Results are shown to be significant at *p* < 0.05. EPO: evening primrose oil; TAM: tamoxifen; φ: is significant from the control group; ≠: is significant from the EPO group; *: is significant from the TAM group; θ: is significant from the combination-1 group.

**Figure 7:**
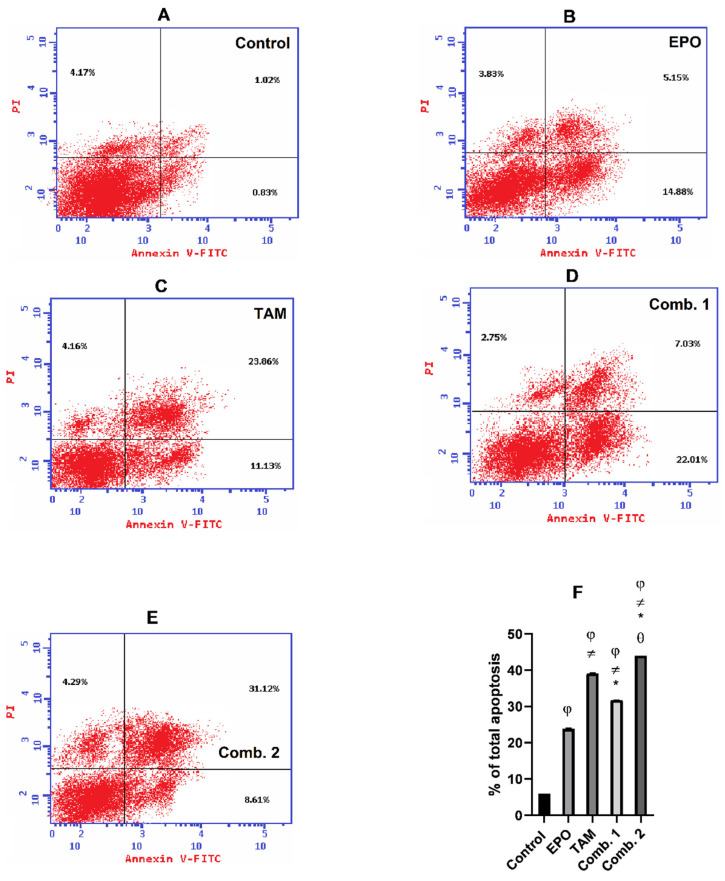
Effect of EPO and TAM, alone or in combination, on apoptosis in MDA-MB-231 cells: (**A**–**E**) Dot-plots from flow-cytometric analysis of cell apoptosis using annexin V-FITC/PI dual-staining in MDA-MB-231 cells. Cells in the upper-left quadrant are necrotic, cells in the upper-right quadrant have late apoptosis, the lower-left quadrant has normal living cells, and the cells in the lower-right quadrant are early apoptotic cells; (**F**) the bar chart describes the total percentage of apoptosis in MDA-MB-231. Results are shown to be significant at *p* < 0.05. EPO: evening primrose oil; TAM: tamoxifen; φ: is significant from the control group; ≠: is significant from the EPO group; *: is significant from the TAM group; θ: is significant from the combination-1 group.

To determine the growth inhibitory effect of TAM and EPO, the percentage of cells undergoing apoptosis upon treatment was detected by flow cytometry. As shown in Figure 6, the percentage of apoptotic cells in the MCF-7 cell line were in the combination-2 group (49.03%), combination-1 group (37.92%), TAM group (42.18%), EPO group (25.19%), and control group (3.92%). Meanwhile, (Figure 7) demonstrates that the percentage of apoptotic cells in the MDA-MB-231 cell line were in the combination-2 group (44.02%), combination-1 group (31.79%), TAM group (39.15%), EPO group (23.86%), and control group (2.57%).

Although apoptosis was induced in all the treated groups, the results of annexin V- FITC/PI dual-staining clearly demonstrate that the combination-2 group triggered the highest induction of apoptosis.

### 3.6. Effect of TAM and EPO, Alone or in Combination, on Cell Cycle

To identify whether the growth inhibitory effects of EPO and TAM are due to the blockage in cell-cycle progression, we evaluated their potential roles on the cell cycle using flow cytometry, and the percentages of cells in the G0/G1, S, andG2⁄M phases were calculated.

**Figure 8:**
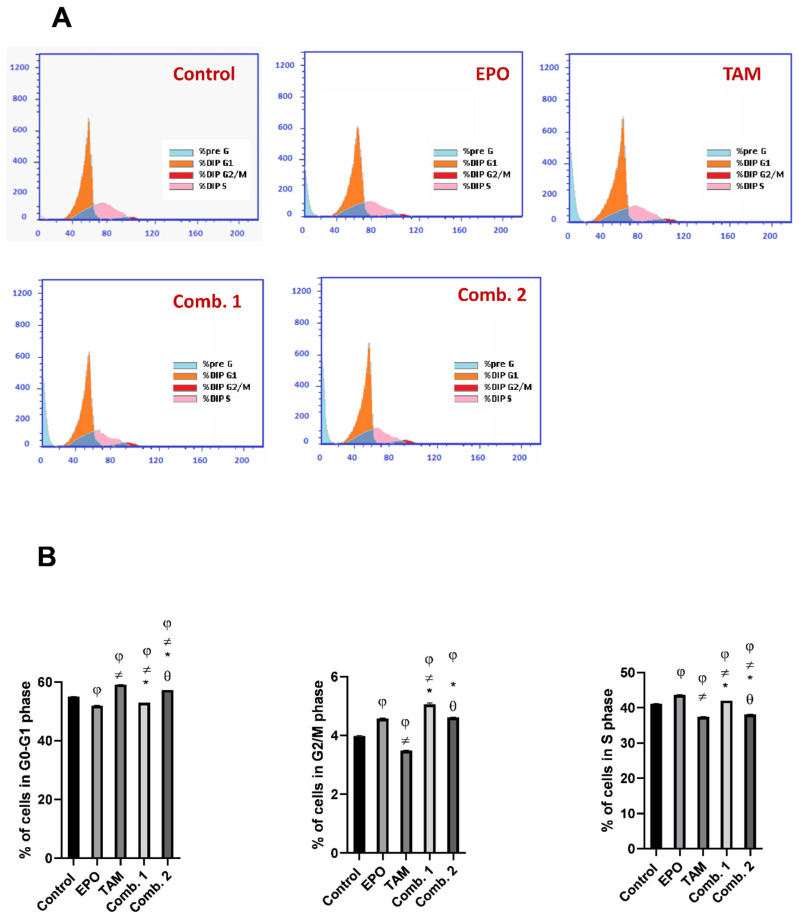
Effect of EPO and TAM, alone or in combination, on cell cycle in MCF-7 cells: (**A**) cell-cycle histograms of different groups; (**B**) graphical presentation of % of cells in G0/G1, S, and G2/M cell-cycle phases. Results are shown to be significant at *p* <0.05. EPO: evening primrose oil; TAM: tamoxifen; φ: is significant from the control group; ≠: is significant from the EPO group; *: is significant from the TAM group; θ: is significant from the combination-1 group.

As demonstrated in (Figure 8), the cell-cycle progression of MCF-7 cells was arrested at the S phase (EPO group), G1 phase (TAM group), G2 phase (combination-1 group), and G1/S phase (combination-2 group). The percentages of cells accumulating in S phase were 36.88% (Control), 43.61% (EPO), 37.41% (TAM), 42.02% (combination-1), and 38.11% (combination-2), while the percentages in the G2/M phase were 6.74% (Control), 4.59% (EPO), 3.47% (TAM), 5.07% (combination-1), and 4.61% (combination-2). Furthermore, the G0/G1 phase of cell-cycle distribution was 56.38% (Control), 51.8% (EPO), 59.12% (TAM), 52.91% (combination-1), and 57.28% (combination-2).

**Figure 9:**
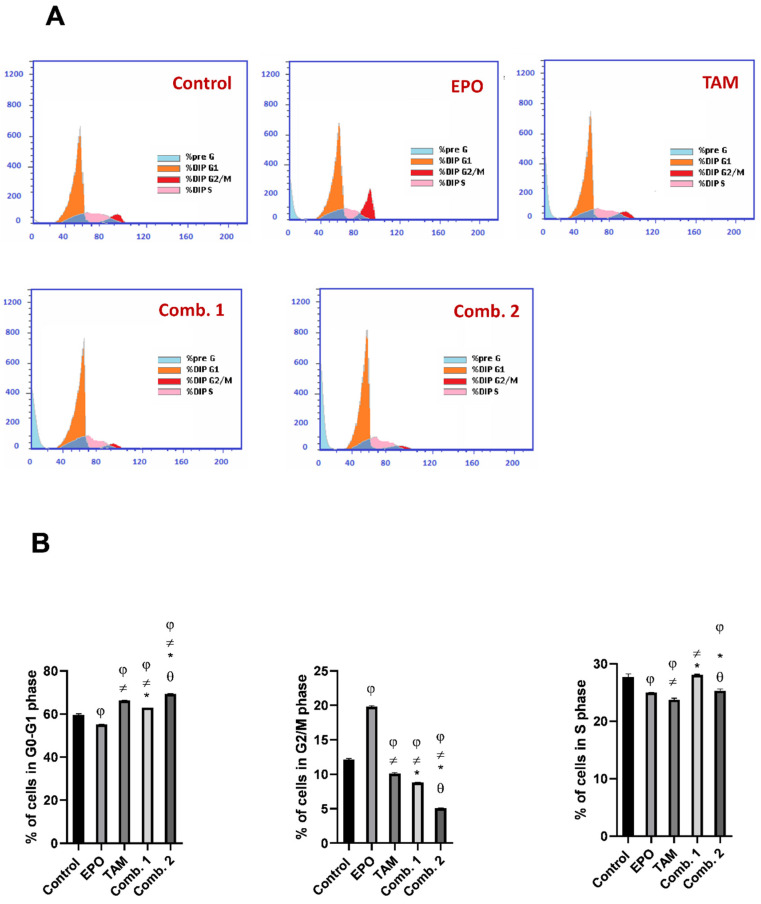
Effect of EPO and TAM, alone or in combination, on cell cycle in MDA-MB231 cells: (**A**) cell-cycle histograms of different groups; (**B**) graphical presentation of % of cells in G0/G1, S, and G2/M cell-cycle phases. Results are shown to be significant at *p* < 0.05. EPO: evening primrose oil; TAM: tamoxifen; φ: is significant from the control group; ≠: is significant from the EPO group; *: is significant from the TAM group; θ: is significant from the combination-1 group.

As shown in (Figure 9), the cell-cycle progression of MDA-MB231 cells was arrested at the G2/M phase (EPO group), G1 phase (TAM group), G1 phase (combination-1 group), and G1 phase (combination-2 group). The percentages of cells accumulating in the S phase were 29.51% (Control), 24.99% (EPO), 23.42% (TAM), 28.16% (combination-1), and 25.48% (combination-2), while the percentage at the G2/M phase were 9.43% (Control), 19.75% (EPO), 10.17% (TAM), 8.82% (combination-1), and 5.11% (combination-2). Furthermore, the G0/G1 phase of cell-cycle distribution was 61.06% (Control), 55.26% (EPO), 66.41% (TAM), 63.02% (combination-1), and 69.41% (combination-2).

## 4. Discussion

TAM is a frequently utilized chemotherapeutic agent in patients with ER-positive BC [18]. Combination therapy has evolved into a comprehensive strategy for cancer treatment. Natural anticancer agents are generally safe, and their combination with cytotoxic agents may have a synergistic therapeutic impact, resulting in decreased chemotherapy dosage, toxicity, and drug resistance [19]. It was suggested that EPO, as a rich source of γ-linolenic acid, supports anti-cancer therapy [14].

This study aimed to determine the anticancer efficacy of EPO, alone or in combination with TAM, in (ER-positive) MCF-7 and (triple-negative) MDA-MB-231 cell lines. Apoptosis, angiogenesis, cell-cycle progression, and cytotoxicity were assessed to determine the anticancer effects of the studied agents.

The results of the present cytotoxicity studies showed significant antiproliferative activity of TAM and EPO on MCF-7 and MDA-MB-231 cell lines. The cytotoxic effect of TAM was dose-dependent, as it showed an effect on MCF-7 cells at a concentration of 0.78 µg/mL, which is a lower dose than was required by the MDA-MB-231 cells (5.92 µg/mL). TAM demonstrated effectiveness against triple-negative MDA-MB-231 cells at a higher dosage, representing heightened TAM toxicity and adverse effects; thus, using the combination to potentiate its effect—which may result in lowering its required therapeutic dose and, therefore, reduce toxicity—might be an excellent approach. EPO cytotoxicity results showed that it affects the cell viability of the MDA-MB-231 cell line in a higher dose than MCF-7 cells. One possible explanation for the difference in antiproliferative activities of TAM and EPO on the two cell lines is their differing ER status, since the MCF-7 line is ER-positive while MDA-MB-231 is ER-negative [20].

Apoptosis is a natural inhibitor of tumorigenesis, and its stimulation in malignant cells is one of many cancer chemopreventive and chemotherapeutic techniques [21]. The present study revealed that the administration of the natural product EPO, alone or in combination with the anticancer drug TAM, induced apoptosis in MCF-7 and MDA-MB-231 cell lines. Apoptosis was evidenced by a significant decrease in the anti-apoptotic Bcl-2 gene expression and a significant increase in the expression of the pro-apoptotic Bax gene. The activity of caspase-3, one of the protease enzymes playing an essential role in apoptosis, also increased after treatment. MCF-7 and MDA-MB-231 treated with the combination of the two drugs showed the highest Bax expression and caspase-3 activity and the lowest Bcl-2 expression, indicating a higher apoptotic activity rate. Further, combination-2 showed the most significant results among all treated groups. These findings were assured by flow-cytometric analysis of cell apoptosis, which revealed the highest apoptotic percentage in combination-2-treated groups in the two cell lines, with a significant difference from other groups. In agreement with our findings, Lewandowska et al. showed that EPO and its major component, Polyunsaturated fatty acids, had anticancer activity [22]. Upon uptake, linoleic acid will be converted into γ-Linolenic acid in the presence of ∆-6 desaturase, then elongated by elongase to become dihomo-γ-linolenic acid. γ-linolenic acid and dihomo-γ-linolenic acid may exert anticancer properties by altering cyclooxygenase metabolism, producing PGE1. It has been demonstrated that they are able to regulate gene and protein expression, disrupting cell-cycle progression; this stimulates reactive oxygen species and caspase activation, cytochrome c liberation, and finally, apoptosis [23,24,25]. Additionally, various studies have discussed the anti-tumor activity of palmitate. Exogenous palmitate promoted cell-cycle arrest, DNA damage, autophagy, and apoptosis in two human endometrial epithelial cell lines [26]; suppressed cell-membrane fluidity and glucose metabolism in hepatocellular carcinoma cells [27]; induced cell-cycle G2/M arrest and promoted apoptosis in human neuroblastoma cells and breast cancer cells [28,29]; induced a different transcription program in breast cancer, reducing expression of HER2 and HER3 [30]; and showed selective toxicity and induced apoptosis in leukemia cell lines [31].

The Bax/Bcl-2 ratio appears to be more essential in guiding the sensitivity to the apoptotic inducer than the expression of each protein alone. If the Bcl-2/Bax mRNA ratio is greater than 1, the cells are vulnerable to pro-apoptotic agents. On the other hand, as the ratio lowers, the cells become more resistant to treatment [32]. In this context, our results revealed an increase in the Bax/Bcl-2 ratio in the combination-2 group compared to all treated groups. These findings suggest that EPO, alone or in combination, is a more potent inducer of apoptosis in MCF-7 cells than in MDA-MB-231 cells. These findings are consistent with previous research indicating that EPO may alter the Bcl-2/Bax mRNA ratio, making prostate cancer cells more susceptible to apoptosis-inducing drugs [33].

Angiogenesis is a multistage, complicated process. Endothelial cell activation, migration, proliferation, and the generation of proteolytic enzymes that degrade the basement membrane, are all critical events in the development of the new blood vessels essential for cancer development and growth [34,35,36]. The present study results showed that the treatment with EPO, TAM, or their combination significantly downregulates the expression of the VEGF gene and decreases its level. These antiangiogenic effects were more potent in combination groups; the most notable effects were shown in the combination-2 group. VEGF is a remarkably angiogenic factor that plays a critical role in tumor angiogenesis. Most solid human tumors, including pulmonary, breast, gastrointestinal, hepatic, ovarian, and intracranial tumors, overexpress VEGF. As a result, blood VEGF levels are regarded as an excellent tumor marker and a very effective anticancer therapeutic target [37]. Therefore, the most antiangiogenic strategies—particularly those used to treat cancer—are based on VEGF suppression [34]. The γ-Linolenic acid content of EPO causes an increase in the expression of the nm-23 metastasis-suppressor gene in cancer cells, which favors the inhibition of angiogenesis, cancer cell migration, and consequently, cancer metastasis. The formation of these changes is also associated with a reduction in the expression of VEGF [23].

Cell-cycle progression and arrest have been thoroughly investigated, and Cyclin D1 has been implicated in the G1/S transition and cell division regulation [38]. Cyclin-D1 overexpression is correlated with malignant tumors [39]. The present study revealed that both drugs, and their combination, significantly decreased cyclin D1 levels in both cell lines; the combination-2 group had the most significant effects. In this context, the flow-cytometric analysis of the cell cycle demonstrated that the cell-cycle progression of MCF-7 and MDA-MB-231 cells was arrested. The arrest of cell-cycle progression in combination-2-treated groups were significant from other treated groups. These findings demonstrate that EPO inhibits MCF-7 and MDA-MB-231 cell proliferation by inducing cell-cycle arrest.

The obtained in vitro results on MCF-7 and MDA-MB-231 cells establish the efficacy of the TAM and EPO combination before clinical trials. Further studies are needed to choose the most effective drug combination that may reduce the therapeutic dose of TAM, minimizing its unwanted side effects.

## 5. Conclusions

The most significant finding of this study was the confirmation of the anticancer activity of the natural product EPO; it potentiated the activity of the anticancer drug TAM against MCF-7 and MDA-MB-231 BC cell lines through the induction of apoptosis, inhibiting angiogenesis and arresting the cell cycle. According to our findings, EPO may be used in combination with tamoxifen to treat breast cancer, since it enhances its efficacy; this may result in a reduction in the required tamoxifen dose, minimizing its toxicity and side effects.

## Figures and Tables

**Table 1:** Primer sequences used on qRT-PCR.

Gene	Forward Primer (5′–3′)	Reverse Primer (5′–3′)
VEGF	AGGAGGAGGGCAGAATCATC	GGCACACAGGATGGCTTGAA
Bax	CCTGTGCACCAAGGTGCCGGAACT	CCACCCTGGTCTTGGATCCAGCCC
Bcl-2	AGGAAGTGAACATTTCGGTGAC	GCTCAGTTCCAGGACCAGGC
β actin	CACCAACTGGGACGACAT	ACAGCCTGGATAGCAACG

**Table 2:** FAMEs detected after derivatization from EPO.

Peak	Retention Time (min)	Name	Area %	Molecular Weight	Molecular Formula	Retention Index
1	7.99	Methyl tetradecanoate (Myristic acid, Methyl ester)	0.23	242	C_15_H_30_O_2_	1725
2	11.55	Methyl hexadecanoate (Palmitic acid, Methyl ester)	40.36	270	C_17_H_34_O_2_	1926
3	14.81	Methyl γ-linolenate (γ-Linolenic acid, methyl ester)	7.32	292	C_19_H_32_O_2_	2092
4	15.77	Methyl linoleate (Linoleic acid, methyl ester)	43.05	294	C_19_H_34_O_2_	2092
5	16.25	Methyl stearate (Stearic acid, Methyl ester)	7.91	298	C_19_H_38_O_2_	2128
6	17.75	6,9-Octadecadienoic acid (E, E), methyl ester	0.72	294	C_19_H_34_O_2_	2350
7	18.07	9,12-Octadecadienoic acid (Z, Z) (Linoleic acid)	0.41	280	C_18_H_32_O_2_	2133

## Data Availability

The data presented in this study are available within the article.
